# Microglial reactivity in brainstem chemosensory nuclei in response to hypercapnia

**DOI:** 10.3389/fphys.2024.1332355

**Published:** 2024-02-27

**Authors:** Jaime Eugenín, Sebastián Beltrán-Castillo, Estefanía Irribarra, Raúl Pulgar-Sepúlveda, Nicolás Abarca, Rommy von Bernhardi

**Affiliations:** ^1^ Facultad de Química y Biología, Universidad de Santiago de Chile, Santiago, Chile; ^2^ Centro Integrativo de Biología y Química Aplicada (CIBQA), Universidad Bernardo O’Higgins, Santiago, Chile; ^3^ Facultad de Odontología y Ciencias de la Rehabilitación, Universidad San Sebastián, Santiago, Chile

**Keywords:** microglia, hypercapnia, inflammatory functional state, CD86, CD206, interleukin 1β, TGFβ

## Abstract

Microglia, the resident immune cells of the CNS, surveil, detect, and respond to various extracellular signals. Depending on the nature of these signals, an integrative microglial response can be triggered, resulting in a phenotypic transformation. Here, we evaluate whether hypercapnia modifies microglia phenotype in brainstem respiratory-related nuclei. Adult C57BL/6 inbred mice were exposed to 10% CO_2_ enriched air (hypercapnia), or pure air (control), for 10 or 30 min and immediately processed for immunohistochemistry to detect the ubiquitous microglia marker, ionized calcium binding adaptor molecule 1 (Iba1). Hypercapnia for thirty, but not 10 min reduced the Iba1 labeling percent coverage in the ventral respiratory column (VRC), raphe nucleus (RN), and nucleus tractus solitarius (NTS) and the number of primary branches in VRC. The morphological changes persisted, at least, for 60 min breathing air after the hypercapnic challenge. No significant changes were observed in Iba1+ cells in the spinal trigeminal nucleus (Sp5) and the hippocampus. In CF-1 outbred mice, 10% CO_2_ followed by 60 min of breathing air, resulted in the reduction of Iba1 labeling percent coverage and the number and length of primary branches in VRC, RN, and NTS. No morphological change was observed in Iba1+ cells in Sp5 and hippocampus. Double immunofluorescence revealed that prolonged hypercapnia increased the expression of CD86, an inflammatory marker for reactive state microglia, in Iba1+ cells in VRC, RN, and NTS, but not in Sp5 and hippocampus in CF-1 mice. By contrast, the expression of CD206, a marker of regulatory state microglia, persisted unmodified. In brainstem, but not in hippocampal microglia cultures, hypercapnia increased the level of IL1β, but not that of TGFβ measured by ELISA. Our results show that microglia from respiratory-related chemosensory nuclei, are reactive to prolonged hypercapnia acquiring an inflammatory-like phenotype.

## Introduction

Microglia originate from mesodermal myeloid precursor cells in the yolk sac, which differentiate in tissue-resident macrophages. These primitive macrophages, after differentiation and migration, become resident microglia (Thion and Garel, 2020), which constitute the main defense system of the CNS (Tremblay et al., 2011).

“Homeostatic microglia” (Paolicelli et al., 2022) are dynamic cells that surveil systematically the nervous tissue (Nimmerjahn et al., 2005). They make contact with synapses (Kato et al., 2019), monitor synaptic activity, perform synaptic pruning (Mordelt and de Witte, 2023), and therefore, influence synaptic formation, maturation, and plasticity (Sierra et al., 2010; Miyamoto et al., 2016; Wolf et al., 2017; Kato et al., 2019). When homeostatic microglia detect signals indicative of autoimmune, infectious, ischemic, traumatic, or toxic damage (Rivest, 2009) they trigger an integrative response with the goal of maintaining brain homeostasis (Crain et al., 2013; Paolicelli et al., 2022). In this integrative response, homeostatic microglia switch into reactive microglia, showing modification of their morphology and functional properties (Wolf et al., 2017; Paolicelli et al., 2022). Thus, homeostatic microglia, characterized by small somata, and very thin, long, and arborized branches, become reactive microglia, which show enlarged somata and reduced number and length of branches with scarce arborization (Crain et al., 2013; Doorn et al., 2014; Wolf et al., 2017; Lier et al., 2021; Paolicelli et al., 2022). In an extreme of the multidimensional reactive state are found microglia with amoeboid shape characterized by somata that look swelled with few thick if any branch (Crain et al., 2013; Doorn et al., 2014; Wolf et al., 2017; Lier et al., 2021; Paolicelli et al., 2022).

Reactive microglia can acquire an inflammatory, and even a cytotoxic functionality (Wolf et al., 2017; Jurga et al., 2020; Paolicelli et al., 2022), releasing inflammatory cytokines, increasing phagocytic and degrading capabilities to eliminate potentially harmful endogenous and exogenous compounds (Crain et al., 2013; Paolicelli et al., 2022). In this functional state, microglia, among other features, are characterized by the enhanced expression of cell markers, such as the clusters of differentiation 86, 14, 16, 32 (CD86, CD14, CD16, CD32) (Wolf et al., 2017; Jurga et al., 2020; Paolicelli et al., 2022), and the release of several pro-inflammatory cytokines, such as interleukin 1β (IL1β), interleukin 6 (IL6), tumor necrosis factor α (TNFα), and interferon γ (IFNγ), plus reactive oxygen species (ROS), and nitric oxide (NO) (von Bernhardi and Eugenin, 2004; von Bernhardi, 2007; von Bernhardi et al., 2007; Kettenmann et al., 2011; Tichauer and von Bernhardi, 2012; Welser-Alves and Milner, 2013; von Bernhardi et al., 2015a; von Bernhardi et al., 2015b; Wolf et al., 2017; Hickman et al., 2018; Jurga et al., 2020; Paolicelli et al., 2022; Wang et al., 2023). Alternatively, the switch from a homeostatic microglia to a reactive microglia may lead to the acquisition of a regulatory state (Wolf et al., 2017; Jurga et al., 2020; Paolicelli et al., 2022). Reactive regulatory microglia has been characterized by the upregulation of surface molecules such as the clusters of differentiation 206, 32, 64, and 163 (CD206, CD32, CD64, and CD163, respectively), arginase 1 (Arg1) (Wolf et al., 2017; Jurga et al., 2020; Paolicelli et al., 2022; Wang et al., 2023), and the release of anti-inflammatory cytokines and growth factors that promote neuroprotection, such as interleukin 10 (IL10), insulin-like growth factor 1 (IGF1), and transforming growth factor β (TGFβ) (Crain et al., 2013; Wolf et al., 2017; Jurga et al., 2020; Paolicelli et al., 2022; Wang et al., 2023).

Few research addresses whether increased levels of CO_2_ (hypercapnia) can induce the switch from homeostatic microglia into reactive microglia. In fact, only one work has demonstrated morphological changes of microglia induced by hypercapnia (Vollmer et al., 2016). When mice were exposed to air enriched with 5% CO_2_ for 10 min (Vollmer et al., 2016), microglia in the subfornical organ of the mouse hypothalamus showed increased somata size and reduced processes length, consistent with microglia reactivity-in terms of the suggested nomenclature (Paolicelli et al., 2022) replacing “microglia activation”-. Surprisingly, hypercapnia affected only microglia in the subfornical organ but not in other circunventricular organs (Vollmer et al., 2016).

The few additional attempts to reveal microglial reactivity to hypercapnia in other brain regions have been unsuccessful. Thus, breathing 5% CO_2_ enriched air for 20 min was unable to trigger, in adult rats, microglia reactivity in the paraventricular nucleus of the hypothalamus and in brainstem respiratory-related nuclei, such as the locus coeruleus, caudal medullary raphe, and the caudal part of the nucleus tractus solitarius (Marques et al., 2021). Perhaps, in more demanding conditions, microglia reactivity to hypercapnia could be revealed in brainstem respiratory-related nuclei.

Here, we address whether microglial phenotype in brainstem respiratory nuclei can be modified in adult conscious mice by a more prolonged and intense hypercapnic stimulation than that used in previous studies (Vollmer et al., 2016; Marques et al., 2021). Since inhalation of 5% CO_2_ enriched air for 20 min was unable to trigger microglia reactivity in respiratory-related brainstem nuclei (Marques et al., 2021), we exposed mice to 10% CO_2_ in air for 10 and 30 min. It is worth to note that 10% CO_2_ in air mimicks PaCO_2_ levels of 60–80 mmHg, which can be observed in human and rodent physiological conditions (Laszlo et al., 1971; Hayashi et al., 1982; Campbell, 2001; Loeppky et al., 2006) as well as in human pathological conditions (Schafer et al., 1999; Lamon et al., 2012; Shah et al., 2021).

We show here that prolonged intense hypercapnia induces microglia reactivity in several brainstem chemosensory nuclei, but not in other brain areas. Furthermore, assessing expression of markers for the functional state of reactive microglia and the profile of cytokines released in tissue culture, we obtained results suggesting that hypercapnia transforms microglia from a homeostatic into a reactive microglia with inflammatory phenotype.

## Methods

In this article, we adhere to the standardize nomenclature suggested by Paolicelli et al. (2022) to describe microglial functional states (Paolicelli et al., 2022). Bioethical considerations: Experiments were carried out in accordance with the Institute for Laboratory Animal Research (ILAR) Guide for the Care and Use of Laboratory Animals, with the approval of the Bioethics Committee of the Agencia Nacional de Investigación y Desarrollo de Chile (ANID) and the Bioethics Committee of the Universidad de Santiago de Chile (Bioethical inform #180/2021).

**Animals**: Experiments were performed in healthy non-medicated CF-1 newborns at postnatal days 0–2 (P0-P2) and adult CF-1 and C57BL/6 background mice. C57BL/6 correspond to MaFIA-eGFP mice (monocyte-macrophages expressing eGFP and a suicide gene under control of the *c-fms* promoter) in control conditions (Burnett et al., 2004).

C57BL/6 mice are inbred mice, having less genetic variability compared with outbred mice. The genetic homogeneity could lead to a lower within-strain phenotypic variability (Tuttle et al., 2018), which, theoretically, has the practical and ethical advantages of requiring a lower number of inbred mice, compared with outbred mice, to get a defined statistical power. For that reason, we initially explored the reliable hypercapnic stimuli to produce morphological changes in microglia in C57BL/6 mice. Once we established an adequate hypercapnic test protocol, we evaluated whether results can be replicated in CF-1 outbred mouse strains, because CF-1 mice differ from C57BL/6 mice, not only in genetic heterogeneity, but also in their respiratory traits. C57BL6 mice show irregular respiratory rhythm patterns, with more sights and increased rate and duration of spontaneous apneas in both sleep and wakefulness than outbred mice (Tankersley et al., 1994; Yamauchi et al., 2010). In addition, C57BL/6 mice show disordered breathing when recovering from hypercapnic or hypoxic challenges (Tankersley, 2003; Getsy et al., 2014; Getsy et al., 2021). Although CF-1 mice have been used less extensively than C57BL6 mice in respiratory studies, from the perspective of respiratory traits offer a more regular rhythm during eupnea and hypoxic or hypercapnic challenges (Nielsen et al., 1993; Eugenin et al., 2008; Cerpa et al., 2015; Bravo et al., 2016; Beltran-Castillo et al., 2017; Beltran-Castillo et al., 2020).

In addition, CF-1 outbred mice, because their enhanced genetic diversity compared to C57BL/6, offer a more appropriate background of genetic variability as occurs with the genetic variability on which human diseases are developed.

Newborn mice for each experimental group were obtained at random from at least 3-5 different litters. Adult animals were also chosen at random without exclusion criteria.

### Hypercapnia

**FIGURE 1:**
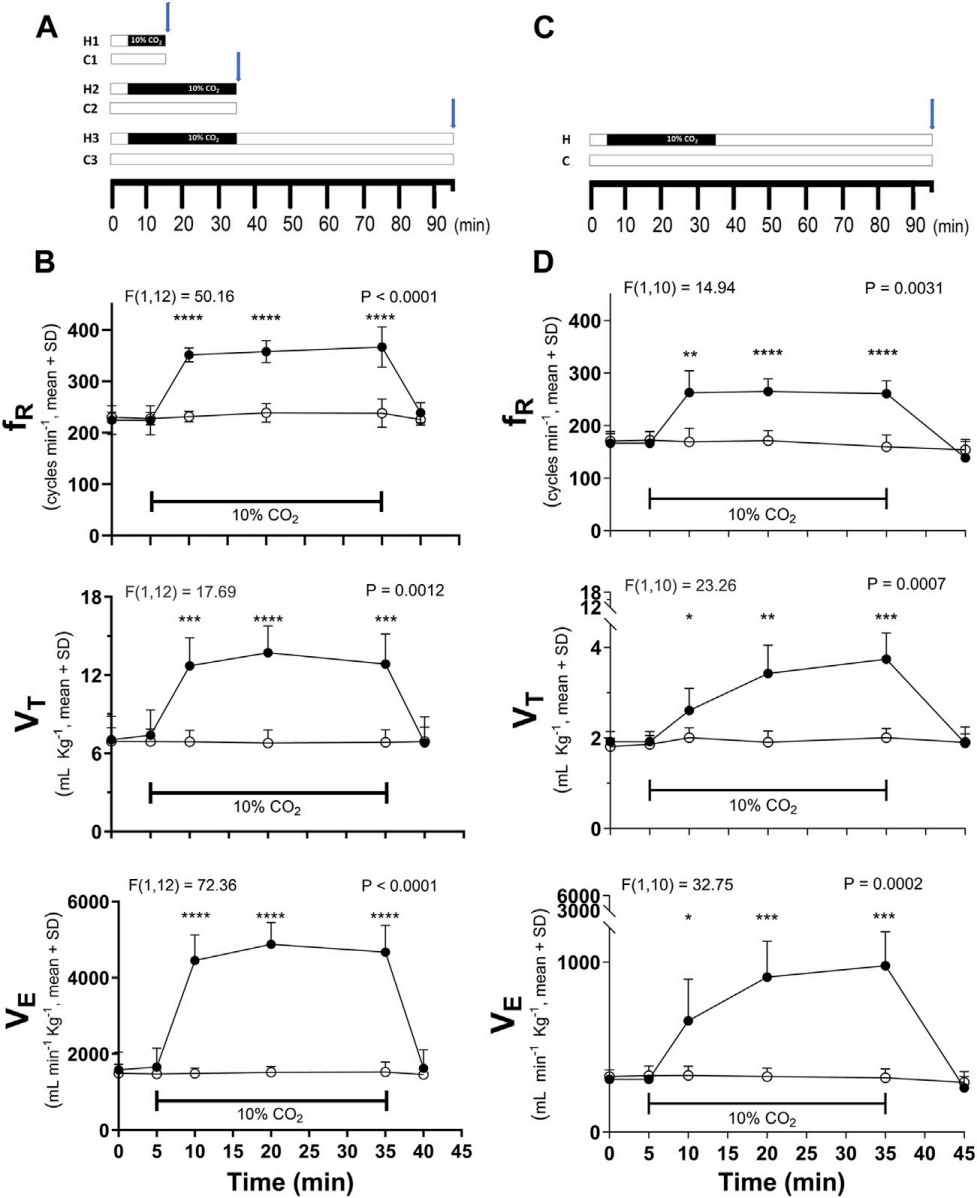
Time course of hypercapnic stimulation and the hypercapnia-induced ventilatory response in respiratory frequency (f_R_), tidal volume (V_T_), and minute volume (V_E_) in C57BL/6 **(A,B)** and CF-1 **(C,D)** mice. **(A)**, scheme of the three stimulation’s protocols used in C57BL/6 mice: hypercapnic test (indicated by the black horizontal bar) for 10 (H1) or 30 (H2) min followed by immediate euthanasia and Iba1 immunolabeling; hypercapnic test for 30 min followed by 60 min breathing air before euthanasia and initiation of immunohistochemistry (H3). C1, C2, and C3, periods in which control mice were breathing air. **(C)**, the H3 protocol was the only protocol used in CF-1 mice. Blue arrows show the time point in which Iba1 immunohistochemistry was initiated. In **(B,D)**, differences in ventilatory variables between control (normocapnia, open circles) and hypercapnia (filled circles) in C57BL/6 (B, *n* = 7 each experimental group) and CF-1 mice (D, *n* = 6 each experimental group). Two-way mixed ANOVA revealed a significant main effect of hypercapnia in both strains (F and respective P are indicated for each respiratory variable); *, **, ***, and ****, *p* < 0.05, *p* < 0.01, *p* < 0.001, and *p* < 0.0001, respectively (Tukey’s *post hoc* test). Symbols and vertical lines, mean ± SD. Horizontal bars indicate periods of hypercapnia, in which treated mice were inhaling air enriched with 10% CO_2_.

As initial approach with C57BL/6 mice, hypercapnic test consisted of a period of basal conditions in which mice breathed air (21% O_2_ in N_2_) for 5 min, followed by a period of hypercapnia in which mice inhaled 10% CO_2_ enriched air for 10 or 30 min, and were immediately processed for immunohistochemistry (Figure 1A). To explore whether these changes were transient or lasted for at least 1 h, after ceasing the hypercapnic challenge, C57BL/6 and CF-1 mice were exposed to air enriched with 10% CO_2_ for 30 min followed by inhalation of pure air for 60 min before performing an Iba-1 immunohistochemistry (Figures 1A, C). Control mice were exposed to air for 10, 30, or 90 min (Figures 1A, C). Unrestricted whole-body plethysmography was used to corroborate the effect of the hypercapnic stimulation (Figures 1B, D).

### Ventilatory recordings

Ventilation was recorded in conscious freely moving adult C57BL/6 and CF1 mice, using whole-body plethysmography under normoxic normocapnia or hypercapnia after 30 min of habituation inside the chamber. Mice were placed in a chamber with a volume of 900 mL and flow of 180 mL min^-1^ and thermoregulated at 28°C–30°C by a temperature controller (FHC Bowdoin, ME, United States). The controller commanded an electric mantle in close contact with the chamber while the internal temperature was monitored with a thermistor (YSI, 44107). Plethysmography recordings were performed when the inflow stopcock was closed, the outflow stopcock was connected to one of the two ports of a differential pressure transducer (spirometer pod ML-311, ADInstruments, New South Wales, Australia), and the plethysmography chamber was hermetically sealed. The signal from the spirometer was digitized at 1 kHz with an A/D acquisition system PowerLab (4/25T model, ADInstruments, New South Wales, Australia) connected to a computer controlled by LabChart 5.0 software (ADInstruments, New South Wales, Australia) for on-line oscilloscope mode display and subsequent data analyses. Calibration was performed by injection of 20, 50, and 100 μL through a small port into the recording chamber with a Hamilton syringe (Hamilton company, Reno, NV). CO_2_ in air was applied from a cylinder containing the final gas mixture of 10% CO_2_, 21% O_2_ balanced in N_2_. The composition of the gas mixture was certified by a reliable gas dealer (Linde gas Chile, Santiago, Chile). Considering the input flow and the volume of the chamber, the time for complete replacement of the air inside the chamber was 5 min, the first time in which the ventilatory variables were measured during hypercapnia. The gas mixture was humidified, and the flow pressure maintained at 5 cm H_2_O by passing gases through the tip of a tube submerged 5 cm below the water surface in a bottle with its output connected to the entrance of the chamber. The administration of pure air through this system did not evoke consistent changes in the amplitude or frequency of respiratory rhythm.

Ventilatory variables were determined *in vivo* around the following time points: a. immediately after the initiation of recording (time 0, basal condition), b. immediately before the beginning of the hypercapnic challenge (time 5, basal condition), c. at 5, d. 15, and e. 30 min of hypercapnia (times 10, 20, and 35 in Figures 1B, D), and f. after 10 min of recovery (time 45, Figures 1B, D); ventilatory variables were estimated from 10 consecutive cycles around these time points in which mice breathed regularly for at least 30s without movement artifacts. If these conditions were not satisfied for a specific time point, then the variable measurement was performed in the closest period at the vicinity of that time point.

### Immunohistochemistry and immunofluorescence

After ventilatory analysis and following the scheme in Figures 1A, C, adult mice were anaesthetized with 2%–3% isoflurane and perfused transcardially with 30 mL of phosphate buffered saline (PBS: 137 mM NaCl; 10 mM Na_2_HPO4; 2.7 mM KCL; 1.8 mM KH_2_PO_4_; pH 7.4) followed by 30–40 mL of fixative (4% paraformaldehyde in PBS). Brains were extracted, immersed in fixative for 24 h, transferred to 10% sucrose for 12 h, followed by 30% sucrose at 4°C for 24 h. Serial 40 μm thick coronal sections of the brainstem and hippocampus were obtained with a CM1510 Leica cryostat. Free-floating sections were placed into PBS filled wells of a 12 well plate. Samples were washed several times in PBS, incubated in bleaching solution (50 mM ammonium chloride in PBS) for 30 min, washed again 4 times for 5 min with PBS, and incubated in blocking solution (3% BSA, 0.1% Triton X-100, 0.01% thimerosal in PBS, pH 7.4) for 1.5 h.

Microglia were identified by immunolabeling Iba1, a 17-kDa protein with expression restricted to microglia/macrophages, (1:1000 polyclonal IgG rabbit anti-Iba1; cat number 019–19741, FUJIFILM Wako Chemical Corporation, United States). Detection was achieved by biotinylated IgG secondary antibody, VECTASTAIN^®^ Elite ABC peroxidase kit (PK-6100), and DAB substrate kit (SK-4100); DAB reaction was allowed to run for 5 min; 0.01% H_2_O_2_, and 0.01% NiCl_2_ for 5 min were used to intensify the dark blue reaction product. Elapsed times for processing all sections from the different conditions and brain regions were the same. Sections from hypercapnia and control trials were run paired.

Images were acquired with a ×20 objective. Characterization of Iba1+ staining on the VRC, RN, NTS, Sp5, and CA1 of hippocampus were quantified as the Iba1 labeling percent coverage to reveal the percentage of pixels Iba1+ in a given photomicrograph, the number of Iba+ cells, their cell body area, and the number of their primary processes using the ImageJ program (ImageJ, NIH) following guidelines from the literature (Green et al., 2022). Morphological variables were determined only in microglia showing their somata with uninterrupted branches at the plane levels.

**FIGURE 2:**
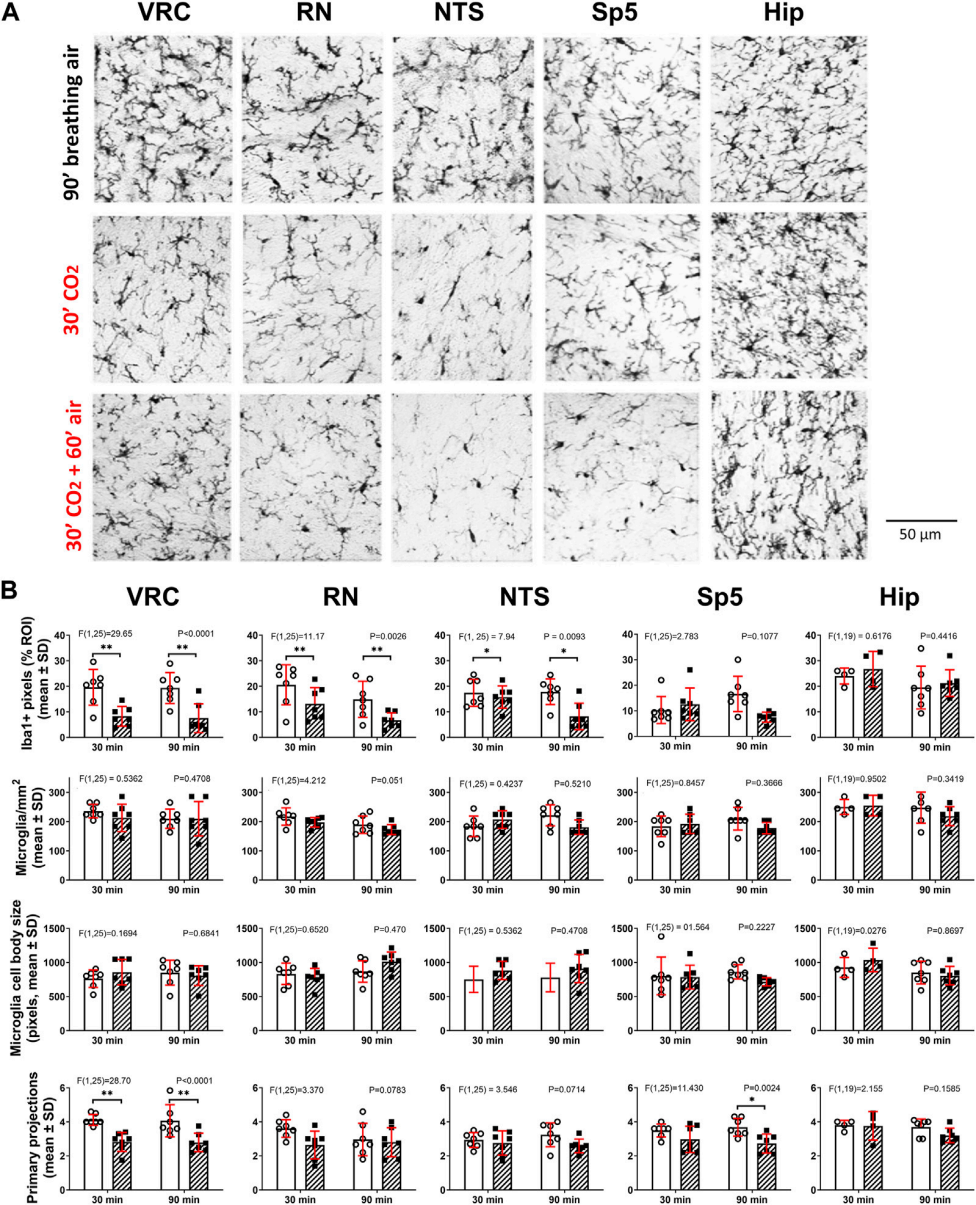
Hypercapnia modifies microglia phenotype in adult C57BL/6 mice. **(A)**, Euthanasia and Iba1 immunohistochemistry were performed immediately (second row, 30′ CO_2_, *n* = 7) or 60 min (third row, 30′CO_2_ + 60′air, *n* = 7) after the end of the hypercapnia stimulation (spontaneous breathing of air with 10% CO_2_ for 30 min). Immunohistochemistry in controls were performed after 30 (*n* = 7, not shown) and 90 min (first row, 90′breathing air, *n* = 7) of spontaneous breathing of air. Iba1+ cells were analyzed in ventral respiratory column (VRC), raphe nucleus (RN), nucleus tractus solitarius (NTS), spinal trigeminal nucleus (Sp5), and in CA1 of hippocampus. Bar, 50 µm. **(B)**, two-way ANOVA followed by Tukey´s *post hoc* test revealed that in both stimulation protocols (30′hypercapnia followed by 0 and 60 min of breathing air) the Iba1 labeling percent coverage (the ratio between the number of Iba1+ pixels and the total number of pixels in ROI, expressed as percentage), was reduced in hypercapnia treated mice in comparison to controls in VRC, RN, and NTS, but unmodified in Sp5 and CA1 of hippocampus. Likewise, two-way ANOVA followed by Tukey´s *post hoc* test revealed the hypercapnia-induced reduction in the number of primary processes in VRC with both stimulation protocols. No significant changes in the number of microglia per mm^2^ and the size of somata of microglia were detected in all five nuclei. Bars and vertical lines, mean ± SD. Control (open bars and circles), exposed to hypercapnia (hatched bars and filled squares). F-value and P are indicated for each analysis; * and **, *p* < 0.05 and *p* < 0.01, respectively.

**FIGURE 3:**
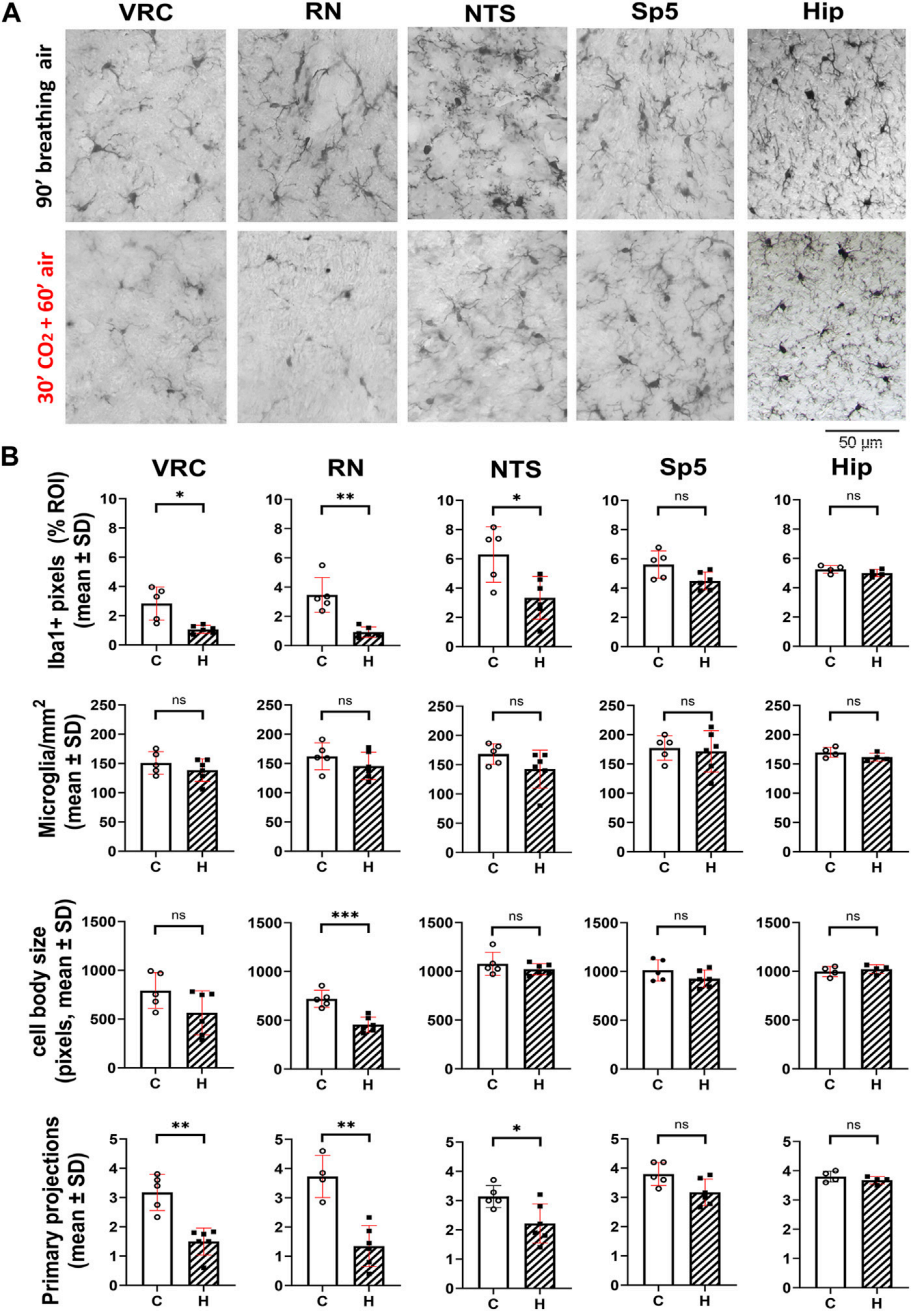
Hypercapnia modifies microglia phenotype in adult CF-1 mice. **(A)**, representative micrographs of Iba1+ cells of adult CF-1 mice after 90 min of spontaneous inhalation of air (first row, control) and after inhalation of 10% CO_2_ in air for 30 min followed by inhalation of pure air for 60 min (second row). Control CF-1 mice show the typical surveillant slender branching morphology of microglia under normoxic normocapnia. As observed in C57BL/6 mice, microglia in VRC, RN, and NTS acquire a reactive phenotype in response to hypercapnia, preferentially reducing their arborization. By contrast, no morphological change is appreciated in Sp5 and hippocampus. Bar = 50 μm. **(B)**, in CF-1 mice exposed to hypercapnia, Iba1 labeling percent coverage was reduced respect to controls in VRC, RN, and NTS, but it was unmodified in Sp5 and CA1 of hippocampus. Accordingly, the number of microglia primary projections was also significantly reduced by hypercapnia in these three nuclei, and unmodified in Sp5 and hippocampus. No significant changes in the number of microglia per mm^2^ and the size of microglia somata were detected in all five nuclei, except for RN, in which the size of somata was reduced. Bars and vertical lines, mean ± SD. Control (open bars and circles), exposed to hypercapnia (hatched bars and filled squares); *, **, and *** indicate *p* < 0.05, *p* < 0.01, and *p* < 0.001, respectively. Unpaired *t*-test with Welch’s correction.

A combination of ImageJ tools was used for analysis. In brief, color micrographs were transformed into gray tones (scale 0–255) using Photoshop CS4 (Adobe) followed by filtering of gray tones using ImageJ program (NIH). Nickel intensification of the DAB reaction gave gray tones distributed between 0–75 (close to black), while background gave gray tones very close to white (>150–255) (Cerpa et al., 2015). These distributions were checked and confirmed in control experiments without antibodies, or with DAB without nickel. To get the Iba1 labeling percent coverage, the threshold tool was applied. Iba1+ labeling was estimated in the region of interest (ROI) from the percentage of Iba1+ pixels of the total number of pixels in ROI. To obtain the cell body number and their size, the nearest pixels to the black were selected and particle analysis was done. Microglia somata sizes were estimated as the number of pixels contained in each soma and expressed in arbitrary units. Microglia projections were highlighted and quantified using threshold and skeleton tools. The total number of microglia within each ROI was determined from 3 consecutive individual sections averaged for each animal, and these averages were used to determine means in animal groups. Somata sizes and the number of primary processes were obtained from 10–15 single cells within three different coronal sections of each animal and averaged. These averages were used to determine means in animal groups (Figure 2B; Figure 3B). In CF-1 mice, length of primary branches was determined using the Fiji’s AnalizeSkeleton plugin of ImageJ software.

Sholl Analysis, that is, quantification of the times connected voxels defining the arbor of a defined cell intersected a series of concentric *shells* (circles or spheres) centered in the cell body, was preceded by preprocessing 2D original images using the Fiji’s Microglia Morphology plugin, to generate bitmap images of individual microglia (Clarke et al., 2021). Then, the Sholl analysis was carried out on the bitmaps of isolated microglia from each region by the Fiji’s Sholl Analysis plugin (Ferreira et al., 2014). The first circle was set to ensure the cell body was not counted as an intercept and the following circles were set at intervals of 4 µm. The number of times that the microglial branches intercepted each of the circles was calculated and plotted with the corresponding distance from soma.

To identify the functional state of reactive microglia, we immunodetect CD86 and CD206 surface markers. CD86, also named B7-2, is one out of eleven members of the B7 family. It is a monomeric protein expressed on the surface of several antigen-presenting cells (APC) such as microglia, macrophages, dendritic cells, B and T cells (Darvish et al., 2023), and endothelial cells (Prat et al., 2000; Girvin et al., 2002). Given that, in the brain tissue, CD86 is expressed constitutively and preferentially on microglia and, besides, it is upregulated in inflammatory microglia, it has been used as a marker of inflammatory state of reactive microglia (Jurga et al., 2020; Paolicelli et al., 2022). Another marker, CD206, also called macrophage mannose receptor 1 (MRC-1), is a transmembrane protein with multiple glycan and C-type lectin binding domains, and a cysteine-rich domain at the N-terminus (Cummings, 2022). The mannose receptor is expressed on macrophages, liver sinusoidal endothelial cells, immature dendritic cells, and in some skin cells such as human dermal fibroblasts and keratinocytes (Cummings, 2022). In the brain can be expressed in microglia, astrocytes, and even neurons (Burudi and Regnier-Vigouroux, 2001; Regnier-Vigouroux, 2003). In microglia, CD206 expression increases when cells adopt a regulatory phenotype. Therefore CD206 was used as a marker of regulatory state of reactive microglia.

Immunofluorescence microscopy was used for double labeling for Iba1 and CD86 (IgG monoclonal mouse antibody, sc-28347; Santa Cruz Biotechnology, United States) or for Iba1 and CD206 (IgG monoclonal mouse antibody, sc-58986, Santa Cruz Biotechnology, United States). Antibodies were diluted 1:1000 in 3% BSA blocking solution, incubated overnight at 4 °C. Sections were washed 6 times for 5 min in PBS, and incubated with the corresponding secondary antibodies, Alexa 546-donkey anti mouse IgG and Alexa 488-donkey anti rabbit IgG (Invitrogen, Eugene, OR, United States) 1:2000 in blocking solution at dark and at room temperature for 90 min. After six washes for 5 min with PBS, nuclei were stained with Hoechst 33258 (1:1000, Invitrogen, Eugene, OR, United States) for 10 min. Slices were rinsed 6 times for 5 min with PBS, mounted on slides, with non-fluorescent mounting medium (DAKO, Glostrup, Denmark), and covered with glass coverslips. Preparations were observed in an epifluorescence microscope (Eclipse model E600, Nikon, Japan) equipped with a Nikon DS-Fi1 digital camera and filters: Nikon UV-2E/C (excitation/emission 340–380/435–485 nm), G-2E/C (excitation/emission 528–553/590–650 nm), and B-2E/C (excitation/emission 465–495/515–555 nm). Analyses were performed on both sides of the brainstem and hippocampus. Observation of microglia was performed on circular fields of 250 μm of diameter (0.049 mm^2^) placed on the VRC, NTS, Sp5, or CA1 of hippocampus, and a rectangular field of 500 × 700 μm (0.35 mm^2^) placed on RN. Each field was captured as 2,560 × 1920 pixels area photographs using ImageJ 1.47t (NHI) software (Wayne Rasband, National Institutes of Health, United States of America). The anatomy for each nucleus was assessed with a mouse brain atlas as reference (Paxinos and Franklin, 2007).

To improve the representation of the labeling, confocal micrographs were performed at high magnification. Observation of samples was done in a laser scanning system (LSM 800; Carl Zeiss) mounted on a motorized Axio Observer Z1/7 (Carl Zeiss) microscope equipped with excitation laser at 405, 488, 561 and 640 nm. Images were acquired with an objective LD C-Apochromat ×40, 1.1 (NA), W Korr UV VIS IR using a Z-stack mode with 1 μm interval, controlled by Zen lite (Carl Zeiss) software at excitation/emission wavelengths of 493/517 for channel 1, 353/465 for channel 2, and 280/618 for channel 3.

### Microglia cultures

Mixed astrocytes-microglia cell cultures and enriched microglia cultures, were obtained from the brainstem and hippocampus as described (von Bernhardi et al., 2015a; Eugenin et al., 2016; Cornejo et al., 2018). In brief, P2 CF1 mice were anesthetized with 3% isoflurane (Aesica Queenborough Limited, Kent, UK) and decapitated. Brainstem or hippocampus were rinsed with Ca^2+^/Mg^2+^-free Hank’s balanced salt solution (HBSS) eliminating meninges before tissue was minced and incubated with 0.25% trypsin-EDTA in HBSS at 37°C for 10 min. After mechanical dissociation, cells were seeded in 25 cm^2^ cell culture flasks (one brain per flask) in Dulbecco’s Modified Eagle’s Medium DMEM/F12 supplemented with 10% fetal bovine serum (FBS), 100 U/mL penicillin and 100 μg/mL streptomycin. Mixed glial cell cultures were incubated in a water saturated environment with 5% CO_2_ at 37 °C for 14 days. Microglia were purified by differential adhesion after treatment with 60 mM lidocaine at 37 °C for 10 min, resuspended, and seeded in a 1:1 mixture of supplemented DMEM/F12 and conditioned media (obtained from mixed glial cell cultures, centrifuged at 200 g for 10 min, and kept sterile). After 1 h of seeding, the medium was replaced by fresh supplemented DMEM/F12. This procedure yields highly enriched microglia cultures (over 99% microglia identified by Iba1 immunohistochemistry). Microglia were plated at a density of 1 × 10^5^ cells in 24-well plates for cytokines determination by ELISA.

### Hypercapnic acidosis *in vitro*

Experiments in culture were performed to assess whether hypercapnia can modify the release of cytokines from microglia obtained from different brain areas (hippocampus vs. brainstem). There is evidence from macrophages in culture that increased CO_2_ downregulates LPS-induced release of cytokines at different time courses (West et al., 1996; Wang et al., 2010). The effect on IL1β appears after 15 min of CO_2_ exposure, whereas the effect on TNF requires more than 30 min of CO_2_ exposure (West et al., 1996). Other consideration is the time required to obtain an amount of released cytokine that is detectable. We used 2 h of hypercapnia stimulation followed by 22 h in normocapnia.

The medium of microglia cultures was replaced by artificial cerebrospinal fluid (aCSF) containing (in mM): 125.0 NaCl, 3.0 KCl, 24.0 NaHCO_3_, 1.25 KH_2_PO_4_, 1.0 CaCl_2_, 1.25 MgSO_4_•7H_2_O (Sigma Aldrich, MO, United States), and 30.0 D-glucose (Merck), equilibrated with O_2_/CO_2_ = 95%/5% (pH 7.4). Microglia cultures were exposed to hypercapnic acidosis generated by switching the aCSF gassing from 5% CO_2_ to 10% CO_2_ in oxygen. Bath gassing with 10% CO_2_ reduced pH from 7.4 to 7.2. A typical hypercapnic acidosis challenge began with the incubation of the preparations with aCSF at pH 7.4 for 30 min, followed by incubation with aCSF at pH 7.2 for 2 h, and a period of recovery at basal conditions (pH 7.4) for 22 h.

### ELISA determination of IL1β and TGFβ

Levels of IL1β and TGFβ were determined by ELISA (eBioscience/Affymetrix) 22 h after the hypercapnic challenge. Cell culture supernatants were collected and kept frozen until the assay. For the assay, 100 µL of supernatant was added to the ELISA plate well coated with the capture antibody and incubated at 4°C overnight (ON). In addition to blank wells, a standard curve with no cytokines (negative control) and increasing concentrations of the recombinant cytokine was included in triplicate for quantification. Detection antibodies were incubated at room temperature for 1 h, and the color reaction was developed with avidin–HRP and substrate solution. Absorbency was measured at 450 nm with reference to 570 nm (microplate reader Synergy HT; Biotek Instruments).

### Data analysis

Experiments were performed using blind conditions, that is, who performed the data analysis did not know the animal identity or the experimental conditions.

The instantaneous respiratory rhythm frequency (f_R_) was calculated as the reciprocal value of the cycle duration and expressed in min^-1^. Tidal volume (V_T_) and minute ventilation (V_E_) were normalized by the pup’s weight. V_T_ was expressed in ml kg^-1^ and V_E,_ which was calculated multiplying V_T_ with the respective f_R_, was expressed in ml min^-1^ kg^-1^. Ventilatory variables were determined *in vivo* during the time course of the hypercapnic test as indicated in Figures 1B, D. Values were expressed as the mean ± SD.

Statistics were performed using GraphPad Prism 5. Sample sizes were defined based on previous studies (Eugenin et al., 2008; Cerpa et al., 2015; Bravo et al., 2016; Beltran-Castillo et al., 2017). Statistical differences of the time course of ventilatory variables in presence or absence of 10% CO_2_ were assessed with two-way mixed ANOVA and differences between control and hypercapnia group at each time point by Tukey´s multicomparison *post hoc* test. The statistical significance between control vs. hypercapnia groups for each microglial morphological trait was evaluated with two-tailed Welch’s unequal variances *t*-test, and when analyzing morphological effects immediately vs. 60 min after the hypercapnic challenge, were assessed with two-way ANOVA followed by Tukey´s multicomparison *post hoc* test. In multiple independent groups, ANOVA test followed by the Tukey´s multicomparison *post hoc* test was used. The null hypothesis was rejected if *p* < 0.05.

### Data availability

Most data generated or analyzed during this study are included in this published article. Any additional or raw data are available from the corresponding authors on reasonable request.

## Results

### Ventilatory responses to hypercapnia

Basal values of ventilatory variables (f_R_, V_T_, and V_E_) for C57BL/6 are higher than those in CF-1 mice as illustrated on Table 1. Likewise, the average maximal increases in V_T_, and V_E_ induced by breathing 10% CO_2_ are greater in C57BL/6 than in CF-1 mice (Table 1). The time courses of the ventilatory responses induced by hypercapnia in adult C57BL/6 and CF-1 mice are shown in Figures 1B, D, respectively. Hypercapnia, produced by inhalation of air enriched with 10% CO_2_, increased significantly the f_R_, V_T_, and V_E_ in adult C57BL/6 and CF-1 mice (Figures 1B, D, respectively), confirming that both strains have the capacity of hyperventilate in response to elevated CO2 (Tankersley et al., 1994; Tankersley, 2003; Eugenin et al., 2008; Cerpa et al., 2015; Bravo et al., 2016).

**TABLE 1:** Comparison of ventilatory values between C57BL/6 and CF-1 mice.

Ventilatory variable	C57BL/6 _(mean±SD)_	CF-1 _(mean±SD)_	p, (n)
basal f_R__(cycles min_^-1^_)_	225.7 ± 27.2	169.0 ± 17.2	<0.000001 (*n* = 14)
basal V_T__(ml Kg_^-1^_)_	7.1 ± 0.3	1.8 ± 0.1	<0.000001 (*n* = 14)
basal V_E__(ml min_^-1^_Kg_^-1^_)_	1563.8 ± 126.9	320.6 ± 41.9	<0.000001 (*n* = 14)
∆ f_R__(cycles min_^-1^_)_	142.5 ± 53.6	94.3 ± 23.0	0.059690 (*n* = 7)
∆ V_T__(ml Kg_^-1^_)_	12.8 ± 1.7	1.8 ± 0.4	0.000001 (*n* = 7)
∆ V_E__(ml min_^-1^_Kg_^-1^_)_	3015.7 ± 791.5	573.4 ± 312.4	0.000071 (*n* = 7)

∆ indicates absolute increase of a ventilatory variable calculated by subtracting the basal value to the maximal value of the variable recorded at the end of the hypercapnic challenge. *p*-value analysis by unpaired *t*-test with Welch correction.

In C57BL/6 mice, f_R_, V_T_, and V_E_ reach high values (very close to maximum) already after 5 min of hypercapnia, whereas in CF-1 mice, only fR rapidly increased to a maximal value during hypercapnia, which was maintained at similarly high level during the hypercapnic test (Figure 1B). After 10 min of recovery inhaling pure air, ventilatory variables returned to basal values. Values of ventilatory variables in control mice inhaling pure air for 90 min, were kept close to the basal values (Figures 1B, D).

### Changes in microglia morphology induced by hypercapnia

Microglia in C57BL/6 and CF-1 mice breathing air during normoxic normocapnia showed a ramified phenotype in brainstem and hippocampus (Figures 2A, Figures 3A) coherent with their homeostatic state. A hypercapnic challenge of 10 min of duration in C57BL/6 mice, followed immediately by immunohistochemistry did not reveal any morphological change in brainstem nuclei microglia (data not shown). By contrast, increasing the hypercapnia duration to 30 min of 10% CO_2_ microglia showed signs of morphological reactivity in the ventral respiratory column (VRC), raphe nucleus (RN), and nucleus tractus solitarius (NTS) (Figure 2A). Analysis of morphological features revealed that after 30 min of hypercapnia the Iba1 labeling percent coverage, that is, the percentage of Iba1+ pixels in the field, were reduced in VRC, RN, and NTS, but unmodified in Sp5 and CA1 (Figure 2B). Only in VRC, but not in RN and NTS, the reduction in the Iba1 labeling percent coverage was associated to a significant reduction in the number of primary processes of microglia (Figure 2B). No significant changes in the number of microglia per mm^2^ and the size of somata of microglia were detected in any nuclei.

To evaluate whether hypercapnia-induced morphological changes were transient or persistent, C57BL/6 mice were exposed to 10% CO_2_ for 30 min but the processing for Iba1 immunohistochemistry was done after 60 min breathing air. As illustrated in Figures 2A, B, morphological effects of hypercapnia upon microglia were similar after 0- or 60-min breathing air after the hypercapnic challenge. However, there was an exception, Sp5 after 30 min hypercapnia-and 60 min of breathing air showed a reduction of the number of primary projections, without a concomitant reduction in the Iba1+ labeling percent of coverage as observed in VRC (Figure 2B). After 60 min breathing air after the hypercapnic challenge, no change in the density of microglia and their somata size were detected in any nuclei.

**FIGURE 4:**
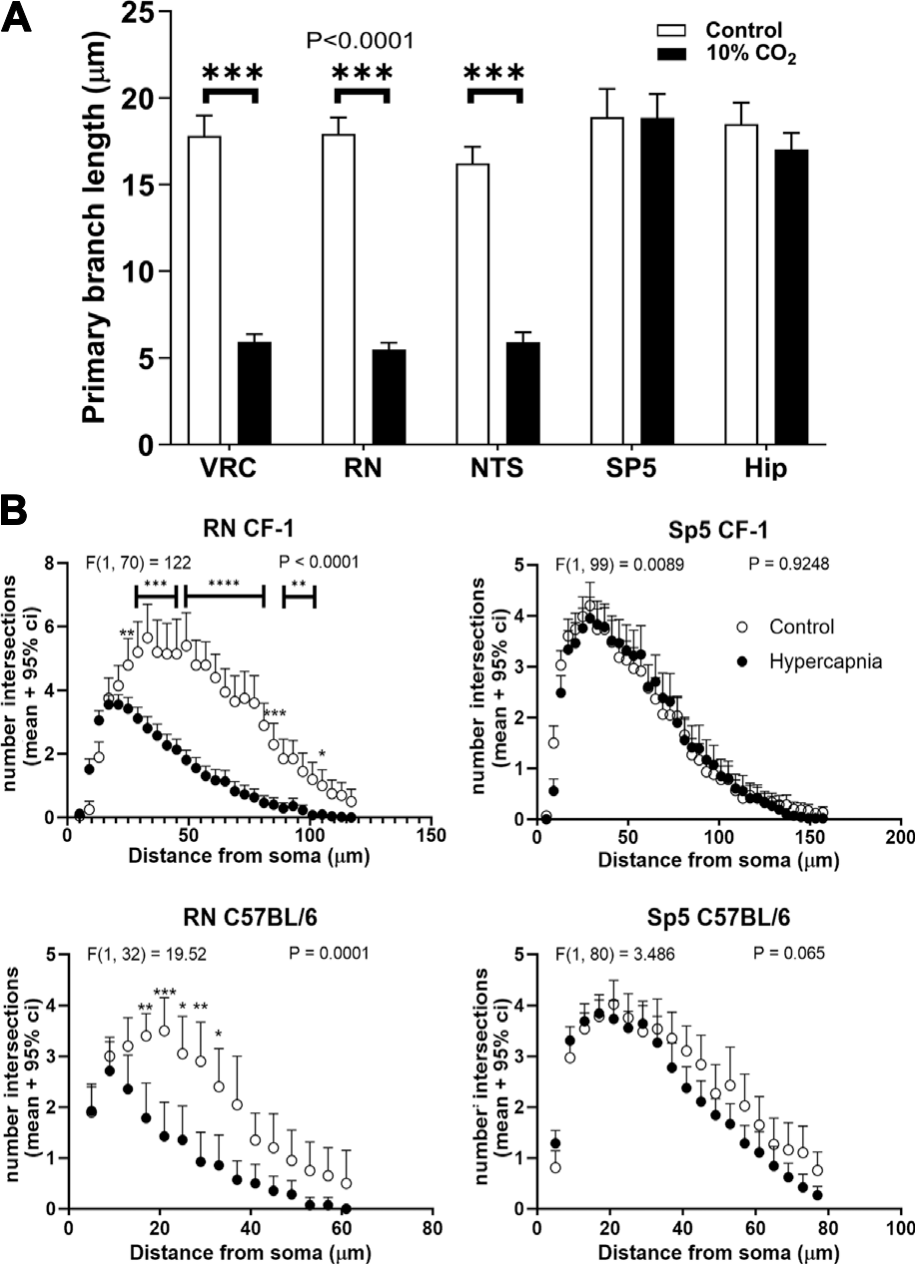
Length and arborization of microglial branches are affected by prolonged hypercapnia in CF-1 mice. **(A)**, comparison of the length of the primary branch of microglia from adult CF-1 mice in different regions: VRC, RN, NTS, Sp5 and hippocampus (Hip). Controls (open bars) mice breathing air for 90 min. Treated CF-1 mice (filled bars) were exposed to 10% CO_2_ in air for 30 min followed by breathing pure air for 60 min before euthanasia and immunohistochemistry. The primary branches of microglia were shortened after hypercapnia in VRC, NTS, and RN, whereas in Sp5 and hippocampus they were unaffected. Bars and vertical lines, mean ± SD (*n* = 15). Two-way ANOVA analysis revealed a significant reduction of primary branch length in VRC, RN, and NTS induced by hypercapnia; df = 1, F (1, 140) = 1823, *p* < 0.0001; ^***^ *p* < 0.001 assessed with *post hoc* Tukeys test for multiple comparisons. **(B)**, Sholl analysis of microglia arborization in a representative brainstem respiratory-related nucleus (RN) and a brainstem non-respiratory nucleus (Sp5) in C57BL/6 and CF-1 mice in control (open circles) and hypercapnia stimulated conditions (filled circles). Two-way mixed ANOVA analysis revealed a significant effect of hypercapnia in the number of intersections of the Sholl analysis in RN. By contrast, Sholl analysis of microglia in Sp5 did not reveal any difference. F-values and P are indicated for each graph; *, **, ***, and ****, *p* < 0.05, *p* < 0.01, *p* < 0.001, and *p* < 0.0001, respectively (Tukey´s *post hoc* test). Symbols and vertical lines, mean +95% confidence interval (CI).

Hypercapnia exposure experiments were performed also in the CF-1 outbred mouse strain, which has a more regular respiratory rhythm and less vulnerability under hypoxic and hypercapnic challenges (Tankersley et al., 1994; Tankersley, 2003; Yamauchi et al., 2010; Getsy et al., 2014; Getsy et al., 2021). CF1 mice microglia, after 60 min past the hypercapnic test transformed from a homeostatic ramified phenotype into a reactive phenotype (Figure 3A). Thus, like our earlier observation in C57BL/6, hypercapnia reduced the Iba1 labeling percent coverage, likely due to the reduction of microglia branching, significantly reducing the number of primary processes in VRC, RN, and NTS (Figure 3B). CF-1 microglia in Sp5 and hippocampus maintained their homeostatic ramified phenotype after hypercapnic test (Figures 3A, B). Concordantly, 30 min hypercapnia plus 60 min breathing air reduced the branch length in VRC, RN, and NTS in CF-1 mice, not affecting the length of branches in Sp5 and hippocampus (Figure 4A). In addition, Sholl analysis of a brainstem respiratory-related nucleus, RN, and a non-respiratory brainstem nucleus, Sp5, show that hypercapnia-induced microglia reactivity affects consistently microglia arborization (Figure 4B) in respiratory-related brainstem nuclei both in C57BL/6 and CF-1 mice.

**FIGURE 5:**
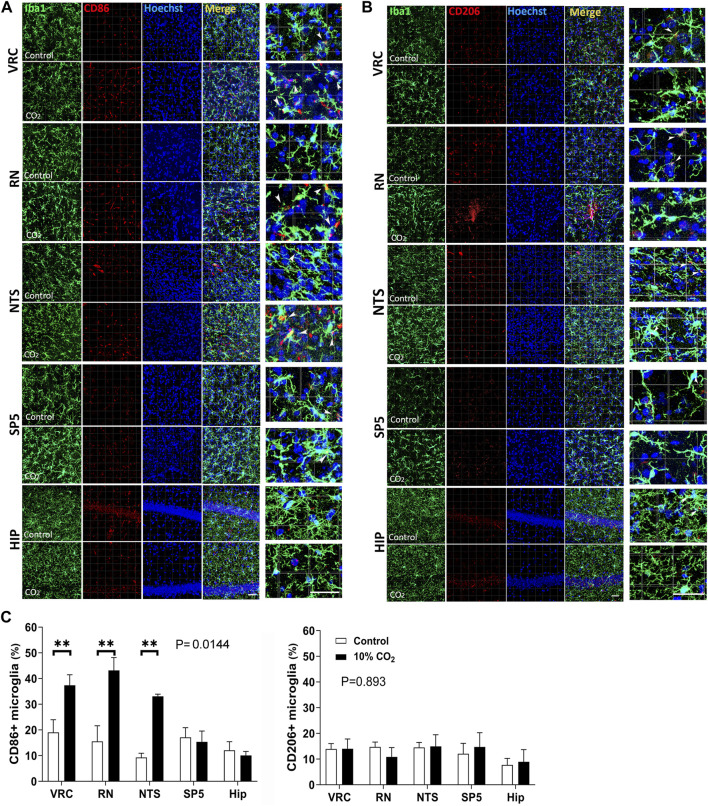
Microglial expression of CD86 (a marker of inflammatory state of reactive microglia) and CD206 (a marker of regulatory state of reactive microglia) in control (Co., normocapnic normoxia) and stimulated (hypercapnic normoxia) CF-1 mice. **(A)**, confocal microscopy of double labeled immunofluorescence detecting Iba1 (green, first column) and CD86 (red, second column) in VRC, NTS, RN, Sp5, and hippocampus of CF-1 mice; Hoechst 33258 in blue, merge, and amplification of a selected field of the merge panel are shown in the third, fourth, and fifth columns, respectively. Note in the amplified merge panel that hypercapnia increases the number of microglia CD86^+^ in VRC, RN, and NTS. Arrow heads indicate Iba1+ CD86^+^ cells. Bars = 100 μm. **(B)**, confocal microscopy of double labeled immunofluorescence detecting Iba1 (green, first column) and CD206 (red, second column) in VRC, NTS, RN, Sp5, and hippocampus of CF-1 mice; Hoechst 33258 in blue, merge, and amplification of selected field of the merge panel are shown in the third, fourth, and fifth columns, respectively. Note in the amplified merge panel that hypercapnia did not elicit significant increase in the density of CD206+ microglia in any of the brainstem nuclei or hippocampus. Arrow heads indicate Iba1+ CD206+ cells. Bars = 100 μm. **(C)**, Analysis of the percentage of microglia expressing CD86 or CD206. Left and right graphs at the bottom, percentage of microglia expressing CD206 and CD86, respectively. Bars and vertical lines, mean ± SD. Control (open bars), exposed to hypercapnia (filled bars). Two-way mixed ANOVA show significant effects of hypercapnia upon the percentage of microglia expressing CD86 in VRC, RN, and NTS (df = 1, F (1,4) = 17.11, *p* = 0.0144); ** indicates *p* < 0.01 between hypercapnia-exposed mice and controls by the Tukey’s multiple comparison *post hoc* test. No significant effects of hypercapnia upon the percentage of microglia expressing CD 206; df = 1, F (1,4) = 0.02076, *p* = 0.893.

To evaluate whether hypercapnia-induced microglial changes were preferentially associated to a reactive microglia inflammatory state, we performed immunofluorescence detection of CD86 (a marker of microglia inflammatory state) and CD206 (a marker of microglia regulatory state) in Iba1+ cells observed after 60 min of being challenged by 30 min of hypercapnia (Figure 5).

As illustrated in Figure 5, confocal micrographs show the distribution and response to hypercapnic conditions of CD86 (5A, C) and CD206 (5B, C). In A, CD86 (in red), shows a granular distribution both intracellular and on the surface of microglial cells. In merge panels, especially with high magnification (last column), is observed that labelling is mainly in microglia cell bodies or close to them in primary processes (white arrows). There is also a few conspicuous blood vessels-like distribution reflecting CD86 presence in endothelial cells. In hippocampal sections there is abundant labelling in the layer of pyramidal neuron cell bodies that is not affected by the hypercapnic condition. Double labeling with the microglia marker Iba1 (in green) indicates that a fraction of CD86 is expressed in microglia. After 30 min hypercapnia the presence of CD86^+^ microglia increased in VRC, RN, and NTS, but not in Sp5 and hippocampus (Figure 5C). In 5B, CD206 (in red), show a similar distribution with that of CD86, although granules are finer. By contrast, hypercapnia had no effect on microglia expression of CD206 in any of the studied regions (Figures 5B, C). The fact that the number of CD86^+^ microglia cells increased with hypercapnia specifically in VRC, RN, and NTS, whereas the number of CD206+ microglia remained unchanged, suggests that hypercapnia induced an inflammatory activation state in respiratory nuclei related microglia (Figure 5).

**FIGURE 6:**
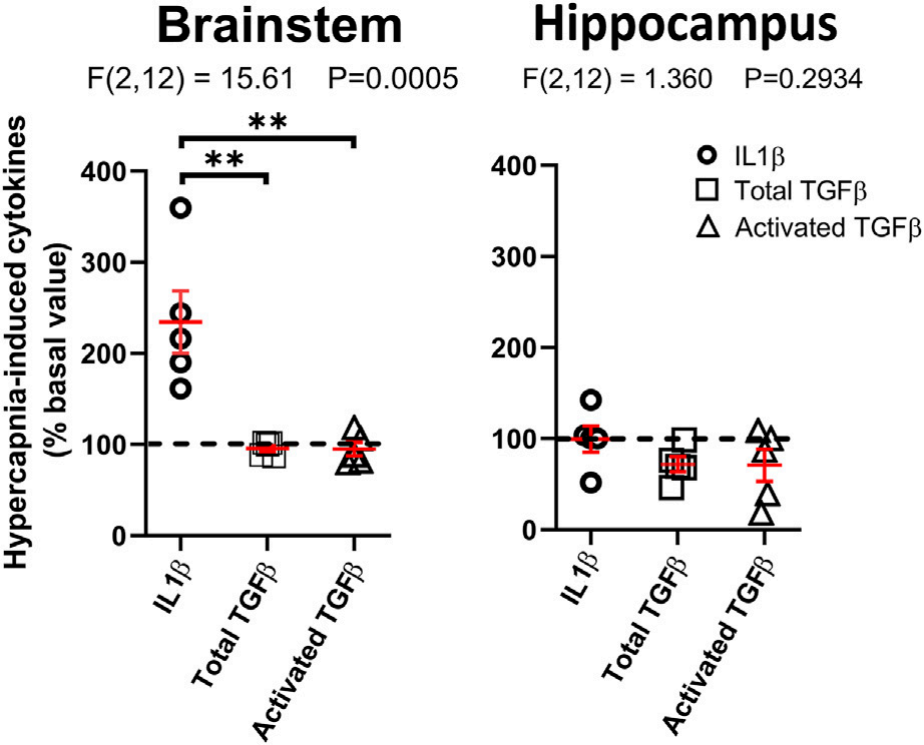
Hypercapnia-induced increase in cytokine level in microglia culture. Brainstem or cortical microglia in culture were stimulated by incubating the cultures in 10% CO_2_ in air for 2 h and return to control condition for additional 22 h. The content of cytokines in the culture medium was assessed by ELISA. The horizontal black dashed line indicates that no change was induced by hypercapnia. Horizontal and vertical red lines correspond to mean ± SEM, respectively. Statistical differences were assessed with one-way ANOVA analysis (*p*-value indicated in each figure); **, *p* < 0.01, Tukey’s multiple comparison *post hoc* test.

The profile of cytokines released by microglia changed by hypercapnic stimulation. Primary cultures of brainstem or hippocampus microglia from CF-1 neonates were gassed with 5% CO_2_/21% O_2_/74% N_2_ (normal conditions) for 30 min, and then switched to 10% CO_2_/21% O_2_/69% N_2_ (hypercapnia) for 2 h followed by 22 h of basal conditions (5% CO_2_/21% O_2_/74% N_2_). ELISA measurement of IL1β and TGFβ, inflammatory-associated and regulatory-associated cytokines, respectively, (Figure 6), show that coherent with the immunofluorescent detection of markers for the functional state of microglia (Figure 5), hypercapnia increased IL1β, but not TGFβ, in primary microglia cultures obtained from caudal medulla. Interestingly, hippocampal microglia cultures did not show significant changes of IL1β and TGFβ, indicating that brainstem but not hippocampal microglia, are susceptible to hypercapnia-induced changes.

## Discussion

Our main results in C57bl/6 and CF1 mouse strains indicate that sustained hypercapnia can transform homeostatic into reactive microglia in brainstem respiratory-related nuclei, such as VRC, RN, and NTS.

The significant changes in morphology occur in the microglial arborization, revealed by the reduction of the Iba1 labeling percent coverage in VRC, RN, and NTS for both strains, the reduction of the number of microglia primary processes in VRC for both strains, and in RN and NTS for CF-1 mice. The respiratory nucleus-specific effect on microglial arborization was corroborated for the reduced length of primary projections in VRC, RN, and NTS in CF-1 mice, and the Sholl analysis performed on RN and Sp5 in both C57BL/6 and CF-1 mice.

Despite of doubling the exposure time to 5% CO_2_, an absence of reactivity to CO_2_ has been reported for rat brainstem microglia (Marques et al., 2021). Comparing the different stimulation protocols, microglia of subfornical organ responded to 5% CO_2_ in air for 10 min (Vollmer et al., 2016), whereas in rat brainstem nuclei, 5% CO_2_ for 20 min (Marques et al., 2021) or, in our study in mice, 10% CO_2_ for 10 min were insufficient to trigger microglia reactivity. To elicit microglia reactivity in VRC, RN, and NTS, a more prolonged and intense stimulus (inhalation of 10% CO_2_ for 30 min) was required. Such value of FiCO_2_ (0.10) generates PaCO_2_ of 60–80 mmHg which can be reached in rebreathing studies in mice and humans (Laszlo et al., 1971; Hayashi et al., 1982; Campbell, 2001; Loeppky et al., 2006) and breathing disorders in humans. For instance, PaCO_2_ in human diseases like Obesity Hypoventilation Syndrome reaches values close to those found in 10% CO_2_ inhaling mice. Patients with mild, moderate, and severe breathing disorders show PaCO_2_ of 46–60, 60–80 and >80 mmHg, respectively (Shah et al., 2021). Other example is Congenital Central Hypoventilation syndrome (CCHS), in which day time PaCO_2_ can be close to 80 mmHg (Lamon et al., 2012) and become much higher, over 100 mmHg during NREM sleep (Schafer et al., 1999). Similar tendencies can be observed in Sleep Apnea Hypopnea Syndrome (Wu et al., 2023).

Microglia reactivity to CO_2_ seems to be a nucleus-specific property in the brainstem and forebrain. In both mouse strains, neither Sp5 nor hippocampus showed microglia modifications induced by hypercapnia. In fact, the unique previous work showing hypercapnia-induced microglia reactivity, of all the murine circumventricular organs evaluated, only microglia within the subfornical organ showed morphological changes in response to hypercapnia (Vollmer et al., 2016). Furthermore, we observed differences in microglia reactivity within the brainstem, among anatomically close nuclei like the respiratory-related nuclei and Sp5. Several studies demonstrate that microglia have regional specificity and differ across brain regions in their density (Lawson et al., 1990), morphology (Lawson et al., 1990; Verdonk et al., 2016; Bottcher et al., 2019; Takagi et al., 2019), gene expression (Tarozzo et al., 2003; Grabert et al., 2016; De Biase et al., 2017; Ewald et al., 2020; Barko et al., 2022), and their proliferative properties (Marshall et al., 2014; Bruttger et al., 2015). Regional specificity of microglia at the level of transcriptome (Ewald et al., 2020; Barko et al., 2022) may be the cause underlying many differences found among brain areas. However, the resolution of transcriptomic studies, in terms of detailed anatomical boundaries, is still poor (Ewald et al., 2020). Within a region like the brainstem, there is not enough information to compare differences in microglial gene expression among brainstem nuclei (Ewald et al., 2020).

In the subfornical organ there is a strong association between microglia reactivity and expression of the microglial channel acid sensor T cell death–associated gene-8 (TDAG8) (Vollmer et al., 2016; Winter et al., 2022). This channel is expressed by microglia in sensory circumventricular organs, preferentially in the subfornical organ. Inhalation of 5% CO_2_ in air in TDAG8^+/+^ mice induces freezing behavior and microglial reactivity, exclusively observed in the subfornical organ. The deletion of TDAG8 gene (TDAG8^−/−^ mice) attenuates the CO_2_ -induced freezing and abolishes microglial cell reactivity in the subfornical organ (Vollmer et al., 2016). Accordingly, increased microglial soma size can be observed 90 min after infusing acidified aCSF restricted to the subfornical organ (Winter et al., 2022). In addition, TDAG8 promoter controlled GFP expression is upregulated in the subfornical organ 24 h after CO_2_ inhalation, but not in the other sensory circumventricular organs. Such selective CO_2_-evoked regulation of TDAG8 promoter activity and microglia reactivity within the subfornical organ, suggests that the sole expression of TDAG8 is not enough to explain the microglial responses to hypercapnia in the subfornical organ.

Preferential expression of intrinsic microglial sensitivity to CO_2_ or H^+^ could underlay nucleus-specific microglial reactivity. Expression of acid-sensing ion channels (ASICS) has been reported in microglia, neurons, and astrocytes from NTS, area postrema, and in cortical microglia cultures (Yu et al., 2015; Lin et al., 2018). Furthermore, expression of voltage-gated proton channel (Hv1) and Na^+^/H^+^ exchanger (NHE) can confer proton sensitivity to microglia (Luo et al., 2021). Voltage-gated proton channels are unique membrane proteins through which only protons can cross the cell membrane (DeCoursey, 2018). In the mouse brain, Hv1 is selectively expressed by microglia and not found in neurons or astrocytes (Wu, 2014). Hv1 activation by pH gradient across the plasmatic membrane, requires to be associated with a strong membrane depolarization for generating an outward H^+^ current (Wu, 2014; Morera et al., 2015). On the other hand, the expression of Na^+^/H^+^ exchanger 1 (NHE1), is not restricted to microglia, although here, the exchanger helps to maintain basal intracellular pH (Liu et al., 2010; Wu et al., 2012; Luo et al., 2021). Up to our knowledge, there are not systematic studies in the brainstem, allowing to correlate the expression of specific channels or transporters in microglia with their reactivity to hypercapnia.

Alternatively, hypercapnia-induced microglia reactivity in selected brainstem nuclei could depend on the hypercapnic activation of neighbor neurons or astrocytes, which could sense changes in PCO_2_ or H^+^ and release neuronal or glial transmitters, respectively (Feldman et al., 2003; Teran et al., 2014; Beltran-Castillo et al., 2017; Gourine and Dale, 2022). Evidence obtained in the RN, NTS, and VRC support the existence of neurons and astrocytes sensitive to CO_2_ and H^+^, resulting in the release of ATP, D-serine, and glutamate, among others factors, which could result in the activation of the neural respiratory network and surrounding cells like microglia (Wang and Richerson, 1999; Severson et al., 2003; Hartzler et al., 2008; Nichols et al., 2008; Gargaglioni et al., 2010; Gourine et al., 2010; Wang et al., 2013; Kumar et al., 2015; Beltran-Castillo et al., 2017; Guyenet et al., 2019; Gourine and Dale, 2022). Regional differences in the capability of neurons and astrocytes to regulate microglia should be matched with the capacity of microglia to sense regulatory signals, which can vary with age and sex (Crain and Watters, 2015). For instance, the expression of purinergic receptors, neuroprotective and pro-inflammatory genes in microglia not only differ among brain regions, but also differ with age and sex in healthy mice (Crain and Watters, 2015).

Additionally, neurons can modulate microglia responses, through several regulatory pathways such as fractalkine, cluster of differentiation 200 (CD200), or TREM2 (Zhang et al., 2018). Similarly, astrocytes can modulate microglial neurotoxicity through a cross-regulation that includes TGFβ and IL1β (Tichauer et al., 2007; Orellana et al., 2013), being the neuroprotective response especially conspicuous in an acidic microenvironment (Uribe-San Martin et al., 2009).

Whether hypercapnia directly modifies microglia or indirectly, through a response mediated by neurons or astrocytes, remains an open question.

### CO_2_ and inflammation

In experimental models of lung injury or ischemia-reperfusion, hypercapnia, mostly in the range of 10%-to-25% CO_2_, has significant impact on alveolar and parenchymal cells (Morales-Quinteros et al., 2019). Among many effects, hypercapnic acidosis antagonizes inflammation and apoptosis, through reduction of neutrophil infiltration, reduction of IL6, IL8, and TNFα levels, and nuclear factor-kappaβ (NFκB) pathway to blunt inflammation, and by blocking p44/p42 MAPK or ASK-1-JNK/p38 pathways to inhibit apoptosis (Contreras et al., 2012; Morales-Quinteros et al., 2019).

Similar hypercapnia effects have been observed in macrophages. In LPS-activated murine alveolar macrophages in culture, hypercapnia -CO_2_ in the range of 2.5–20%- reduced the level of TNFα and increased cytokine-induced neutrophil chemoattractant factor-1 secretion when buffered at pH 7.2 (Lang et al., 2005), whereas in LPS-activated rat peritoneal macrophages in culture -CO_2_ in the range of 5%–80%- reduced TNFα and IL1 levels (West et al., 1996; Lang et al., 2005). Furthermore, in LPS-activated human THP-1 macrophages and human and mouse alveolar macrophages, hypercapnia reduced the expression of TNFα and IL6 but not that of IL10 or IFN-β (Wang et al., 2010); such differential response is compatible with the inhibition of NFκB by CO_2_, since TNFα and IL6 are NFκB-dependent cytokines (Morales-Quinteros et al., 2019). In addition, hypercapnia inhibits phagocytosis in human macrophages (Wang et al., 2010).

All these results point to hypercapnia having a systemic anti-inflammatory role. However, hypercapnia effects appear to be the opposite in the CNS. In TDAG8^−/−^ mice, the level of IL1β in the subfornical organ is reduced, whereas IL1β infusion induced freezing in both TDAG8^−/−^ and TDAG8^+/+^ mice breathing air. Furthermore, intracerebroventricular infusion of IL1RA, the endogenous antagonist of IL1β, reduced significantly the CO_2_-induced freezing, whereas application of IL1β restored freezing behavior decreased by minocycline, an inhibitor of microglia activation (Vollmer et al., 2016). These results sustain the hypothesis that CO_2_, via activation of microglial TDAG8 channel increases the release of IL1 from microglia in the subfornical organ to evoke freezing behavior (Vollmer et al., 2016). In that case, microglia reactivity could be polarized into an inflammatory state. Unfortunately, further attempt to classify the functional state of CO_2_-reactive microglia in the subfornical organ has not been undertaken.

### Microglia reactivity and functional states

Reactive microglia show morpho functional changes, such as enlargement of the cell body, shortening of processes, and increasing phagocytic activity. However, such changes are not pathognomonic of their functional polarization, which can be inflammatory, leading to neurotoxicity, or regulatory (inflammation inhibition, tissue remodeling, angiogenesis, or immune regulation) promoting neuroprotection (Nimmerjahn et al., 2005; Wang et al., 2023). For that reason, as a first approach, we evaluated the expression of key surface markers on microglia, complemented with inflammatory and regulatory cytokines produced by microglia in tissue culture (Crain et al., 2013; Wolf et al., 2017; Jurga et al., 2020; Paolicelli et al., 2022).

To have an insight of the functional state of hypercapnia-induced reactive microglia within specific brain areas, we assessed by confocal microscopy the presence of CD86, a surface marker of inflammatory reactive microglia and CD206, a surface marker of regulatory state of reactive microglia (Crain et al., 2013; Wolf et al., 2017; Jurga et al., 2020; Paolicelli et al., 2022). We found that the percentage of CD86^+^ microglia in CF-1 mice increased after 30 min of 10% CO_2_ followed by 60 min breathing air in VRC, RN, and NTS, whereas CD86 was unchanged in microglia within non-respiratory structures, such as Sp5 and hippocampus. By contrast, the percentage of CD206+ microglia was not modified by the hypercapnic challenge in any of the five nuclei.

Furthermore, in brainstem microglia cultures, 2 h of hypercapnic exposure followed by 22 h of incubation on basal conditions, increased the levels of the inflammatory cytokine IL1β, whereas the level of total and active TGFβ, a regulatory cytokine, were unchanged. The effect of CO_2_ depended on the brain region origin of microglia, with CO_2_ stimulation failing to evoke any response in hippocampal microglia cultures. These results are compatible with our immunolabelling results, indicating that CO_2_-induced microglia reactivity may be functionally polarized towards an inflammatory phenotype.

Hypercapnia in normoxic *in vivo* conditions (5% CO_2_, 21% O_2_, 74% N_2_) for 3 h was unable to increase the expression of IL1β in microglia of adult rat hippocampal CA1 area examined by double immunofluorescence (Ding et al., 2018). Similarly, normoxic hypercapnia (15% CO_2_, 20% O_2_, 65%N_2_) for 24 h did not increase IL1β in BV2 microglial cells in culture (Ding et al., 2018). However, hypercapnia under hypoxic conditions was effective in increasing IL1β both *in vivo* and *in vitro* (Ding et al., 2018). Differences with our *in vitro* results, might be related with the different species (rat vs. mouse), the use of BV2 cell lines instead of primary microglia, distinct stimulation intensity (10% vs. 15% CO_2_) and duration (2 h vs. 24 h), or perhaps different basal microglial functional states (Paolicelli et al., 2022).

### Possible role of microglia reactivity associated to respiratory nuclei

Among several microglia secreted inflammatory factors, IL1β, TNFα, and IL10 has been implied in breathing modulation (Lorea-Hernandez et al., 2016; Giannakopoulou et al., 2019; Pena-Ortega, 2019; Lorea-Hernandez et al., 2020). In urethane-anesthetized adult rats under normoxic conditions, increased level of plasma TNFα increases V_T_ and V_E_, but leave f_R_ unmodified. Whereas i. p injected IL10, in neonatal rats in normoxic normocapnia reduces the f_R_ and increases the V_T_ (Giannakopoulou et al., 2019). Nevertheless, administration of IL10 on rhythmically active medullary slice preparations do not affect neither rhythmicity nor peak amplitude of hypoglossal nerve discharge (Giannakopoulou et al., 2019).

Based on our *in vitro* results, IL1β secreted by microglia in inflammatory state appears to be a good candidate to act on the respiratory network. *In situ* hybridization revealed the presence of IL1β receptor mRNA in several respiratory-related brainstem nuclei in rats (Yabuuchi et al., 1994; Ericsson et al., 1995). Accordingly, an increased expression of c-fos is observed in several brainstem respiratory-related circuits in response to systemic administration of IL1β (Brady et al., 1994). However, the respiratory effect of IL1β depends on the species, preparation, and via of administration. In addition, the complete details about the location of IL1β receptors, whether they are expressed by different cell types, or in specific respiratory nuclei, or whether their effect is nucleus-specific are still unknown. Therefore, with the present state of the art it is not possible to predict the specific respiratory effects of IL1β regardless its release by reactive microglia within the VRC, RN, or NTS. Intraperitoneal IL1β injection reduces f_R_ without modifying V_T_ and V_E_ in conscious rats (Olsson et al., 2003), whereas it reduces V_T_ and V_E_ in mice (Hofstetter and Herlenius, 2005). By contrast, i. v. IL1β injection increases f_R_ in conscious rats (Graff and Gozal, 1999) and i. p. IL1β injection increases the amplitude of respiratory cycle in anesthetized rats (Hocker and Huxtable, 2018). In urethane-anesthetized normoxic rats, i. v. administration of IL1β increases V_T_, f_R_, and V_E_ (Donina et al., 2021).

On the other hand, the respiratory effect of intrathecal IL1β has been explored in anesthetized, tracheostomized, spontaneously breathing rats. The intracerebroventricular injection of human recombinant IL1β under resting condition increases V_E_ and mean inspiratory flow, and reduces the ventilatory response to hypercapnia (Graff and Gozal, 1999; Aleksandrova and Danilova, 2010), suggesting that IL1β contributes to the central control of breathing and chemoreflex sensitivity (Aleksandrova and Danilova, 2010).

Application of IL1β to *in vitro* preparations generates diverse results. In the “*en bloc*” brainstem-spinal cord preparation, IL1β superfusion does not modify fictive respiration (Olsson et al., 2003), whereas IL1β injection in the NTS in the “*en bloc*” preparation, reduces the frequency of fictive respiration (Gresham et al., 2011). Furthermore, in mouse brainstem slices containing the pre-Bötzinger complex, IL1β reduces the amplitude of fictive respiration in a concentration-dependent manner (Lorea-Hernandez et al., 2020).

### Limitations of the experimental approach

In general, a more precise identification of the functional states of microglia may require a broader battery of immunodetected markers and a wider spectrum of cytokines. However, the increased expression of surface inflammatory marker CD86 in microglia exposed to CO_2_ is coherent with the absence of change in expression of CD206 and the enhanced release of the inflammatory cytokine IL1β by microglia in cultures challenged with CO_2_, associated with the null effect on TGFβ release.

One important caveat are the differences between *in vivo* and *in vitro* experiments including the basal microglia functional state in which experiments are performed, given their potential impact on the CO_2_-induced microglial reactivity. Results from *in vitro* experiments should be taken cautiously. However, they provide also compelling evidence on the particularities of microglia associated with nuclei involved in respiratory regulation. Our results show clearly that neonatal microglia obtained from brainstem but not from hippocampus, exposed in culture to an hypercapnic challenge for 2 h followed by incubation in basal conditions for 22 h release increased amounts of IL1β, but not TGFβ. The interpretation of the results should take account that functional states of microglia are dynamic and plastic (Paolicelli et al., 2022) and the results obtained could be transient and not necessarily be maintained on a longer time course but being still relevant for the acute regulation of breathing.

### Summary and translational perspective

Prolonged hypercapnia induces the transformation of homeostatic microglia into reactive microglia in specific brainstem respiratory nuclei of C57BL/6 and CF-1 mice, but not in Sp5 and hippocampal microglia. Immunofluorescence detection of surface inflammatory or regulatory markers to recognize functional states of reactive microglia, as well as ELISA detection of inflammatory (IL1β) and regulatory (TGFβ) cytokines by microglia, revealed that, likely, hypercapnia-induced microglia acquire an inflammatory phenotype. These results, although they do not allow to assure that microglia acquire an inflammatory functional state, they support the suggestion that CO_2_ stimulation induces a reactive response that is restricted to microglia associated to respiratory muclei.

Given that the high levels of CO_2_ selected for the experiments mimics conditions of hypercapnia found in several human breathing disorders, our results provide evidence for new venues searching whether microglia could play a role in human hypoventilation syndromes. Because IL1β can increase basal V_E_, it is reasonable to speculate that in conditions of prolonged high CO_2_ levels, IL1β released by reactive microglia could have a role improving basal alveolar ventilation by increasing the depth of ventilation. On the other hand, it should be also considered that microglial reactivity could lead to an exacerbation of inflammatory damage contributing to the pathophysiology of hypoventilation disorders, particularly, in those conditions in which inflammation is involved in the pathogenesis.

## Data Availability

The raw data supporting the conclusions of this article will be made available by the authors, without undue reservation.
