# Erratum: Microglial reactivity in brainstem chemosensory nuclei in response to hypercapnia

**DOI:** 10.3389/fphys.2024.1404779

**Published:** 2024-03-28

**Authors:** Frontiers Production Office

**Affiliations:** Frontiers Media SA, Lausanne, Switzerland

**Keywords:** microglia, hypercapnia, inflammatory functional state, CD86, CD206, interleukin 1β, TGFβ

Due to a production error, an incorrect **Funding** statement was provided. The correct statement should read:

“The author(s) declare financial support was received for the research, authorship, and/or publication of this article. The current study was supported by grants from the Fondo Nacional del Desarrollo de la Ciencia y Tecnología (FONDECYT REGULAR) 1211359 (JE) and 1221028 (RB), and FONDECYT INICIACIÓN 11230857 (SB-C) from the Agencia Nacional de Investigación y Desarrollo de Chile (ANID).”

The publisher apologizes for this mistake. The original version of this article has been updated.

